# Assessing the role(s) of human touch in immersive entertainment

**DOI:** 10.1177/20416695261437638

**Published:** 2026-04-27

**Authors:** Yang Gao, Charles Spence

**Affiliations:** 1 6396 University of Oxford, Oxford, UK

**Keywords:** physical touch, immersive entertainment, haptic feedback, audience engagement, affective touch

## Abstract

Physical touch plays an important role in various forms of immersive entertainment, influencing emotional engagement, presence, and narrative immersion. In this narrative historical review, we explore the significance of direct interpersonal (social) touch in entertainment contexts. This includes theme parks, immersive theater, participatory performance art, and digitally mediated touch (e.g., haptic feedback in virtual reality [VR], gaming, and film). By analyzing examples such as Disneyland character interactions, Sleep No More, and audience participation in The Rocky Horror Picture Show, we examine how physical touch enhances immersion by helping to break the fourth wall and by fostering audience agency. Additionally, we compare direct human touch with digitally mediated touch (e.g., haptic feedback in VR) and discuss their respective advantages and limitations. While digital touch offers flexibility and scalability, it often lacks the warmth (both literally and metaphorically), texture, and emotional nuance of actual human interaction and can struggle to reproduce the social meaning and attribution that shape affective touch. The review also highlights a number of the key challenges and boundary conditions in implementing physical touch in the context of entertainment, such as cultural variations in norms and comfort around interpersonal touch, privacy/consent concerns, hygiene concerns, and technical constraints. Future research directions include the integration of physical touch with emerging technologies, personalization of touch-based experiences, and the role of multisensory interactions in enhancing immersive storytelling. Understanding the mechanisms and impact of physical touch in entertainment can inform the design of future multisensory experiences, increasing audience engagement and emotional connectivity while minimizing harm and exclusion.

## How to cite this article

Gao, Y., & Spence, C. (2026). Assessing the role(s) of human touch in immersive entertainment. *i–Perception*, *17*(2), 1–18. https://doi.org/10.1177/20416695261437638

## Introduction

Physical touch can shape emotional engagement, presence, and narrative immersion in immersive entertainment. Shanghai Disneyland implemented safety measures during the COVID period in China. That is, the audience and the Disney characters could not touch one another, and the characters could not even touch the gifts brought by the audience.^1^ At that time, the queue to interact with the most popular Disney character, LinaBell, was up to 2 hours long. After COVID restrictions were lifted, Disneyland removed the yellow line, allowing audience members to physically touch, hold hands with, and hug the Disney characters once again. At around the same time, the removal of the yellow line (and the return of physical touch-based interaction) may have contributed to a substantial increase in waiting times for LinaBell. In fact, the queue for LinaBell reportedly increased to more than 5–6 hours.^2^ “In line for six hours, interaction for 2 min” became the normal experience for meeting the Disney character (as of the start of 2025). Typically, the first person to interact with LinaBell each day had to line up outside Disneyland by 3–4 a.m. (with the park usually opening at 9 a.m.). Nevertheless, some visitors were moved to tears when they finally got to interact with the characters (Pan & Wang, 2023; Zou, 2023).

While it's undeniable that Disney's storytelling plays a significant role in how people feel about the characters, the demand for physical interaction, especially after COVID, can, at least in part, be interpreted in terms of the concept of “touch hunger” (a term introduced by Tiffany Field to describe the fundamental human need for physical touch, which is crucial for psychological and cognitive well-being; Field, 2014; Gallace & Spence, 2016; Spence, 2021a). Other factors (e.g., character attachment and narrative association) may also contribute to the demand for touch-based interaction. The exceedingly long queues may also indicate positive consumer contagion, according to which consumers place a higher value on objects, or in this case, experiences, that have been “touched” by a highly attractive or desirable entity (Argo et al., 2006, 2008). In this context, the Disney character acts as the “attractive other,” and physical contact allows for a perceived transfer of some of the character's magic and positive attributes onto the fan. As such, the value of the interaction may come not just from the physical sensation of touch itself but also from the symbolic significance of being in contact with the character. It is a moment of being “touched by” the Disney magic, transforming into an emotional sensory experience that can be interpreted as an “acquisition” of a piece of the Disney narrative. This makes the brief contact valuable and justifies the long wait. This physical interaction between the fan and a character raises several critical questions that include: Who initiates the touch, and how does this influence immersion? How powerful is physical touch in terms of enhancing immersive entertainment, and what can it offer that its emerging digital counterparts cannot?

These digital counterparts often take the form of digitally mediated touch, whereby physical sensations are transmitted remotely. For example, the *inTouch* system, developed at MIT almost 30 years ago (Brave & Dahley, 1997), allows for remote interaction via synchronized rollers, with initial user feedback describing the shared manipulation as “playful” and most applicable to “intimate relationships”.

Over the course of several recent reviews, we have examined the role of digital touch in entertainment, including film (Spence & Gao, 2024a, 2024b), games (Gao & Spence, 2025b), and virtual reality (VR) (Gao & Spence, 2025a), focusing primarily on the applications of digital touch. In this narrative historical review (see Ferrari, 2015; Furley & Goldschmied, 2021, on the strengths of narrative reviews in certain contexts), we focus instead on physical touch in various immersive entertainment media. We highlight the similarities and differences between direct, physical touch, and digitally mediated touch, especially focusing on agency, consent, social meaning, and narrative interpretability.

In this review, the term “immersive entertainment” is used to refer to live and mediated experiences that aim to increase a sense of “being in” the story world and/or active audience involvement (e.g., theme parks, immersive theater, VR, gaming, and more). The distinction between digital touch, such as haptic feedback in gaming and VR, and direct physical human touch, as seen in immersive theater and theme parks, is likely to significantly impact user experience and emotional engagement.

Additionally, some forms of touch in the context of live performances and installations fall into an intermediate category, which might be termed “mediated direct touch,” whereby a physically present actor or performer induces some form of tactile stimulation in the viewer without direct skin-to-skin contact. Such mediated touch can involve tools, wearable devices, or even wind effects designed to create an intentional tactile experience (Hayes & Rajko, 2017; Kontogeorgakopoulos, 2023; Papachatzaki, 2019). Here, mediated direct touch is considered a form of physical, copresent interpersonal touch (immediate and social) that does not involve skin-to-skin contact.^3^ This review therefore explores the distinct roles that physical touch (be it direct or mediated) plays in shaping the audience experience and examines what it can provide that digital touch often cannot.^4^

The review is structured as follows: first, we explore the various dimensions of physical touch in breaking the “fourth wall” and fostering emotional connection. Next, we discuss the distinction between performer-initiated and audience-initiated touch regarding agency. Subsequently, the limitations and cultural considerations of incorporating touch are examined. Finally, we discuss several directions for future research in immersive entertainment, exploring the integration of new technologies and personalization of touch-based experiences. Of necessity, much of the real-world evidence discussed here (e.g., queue length, popularity) is correlational in nature and should thus be interpreted accordingly.

Before proceeding, it is important to establish clear definitions for the core concepts in the review. For the purposes of this paper, “immersion” refers to the technological capacity of a system to deliver an inclusive, extensive, surrounding, and vivid illusion of reality to the sense of a participant (Slater & Wilbur, 1997). “Presence” is the subjective experience of being in one place or environment, even when one is physically situated in another (Witmer & Singer, 1998). These subjective variables are most commonly assessed using validated self-report questionnaires. For example, presence is often measured using questionnaires developed for immersive/VR media (e.g., the Presence Questionnaire, PQ; Witmer & Singer, 1998). Immersion is often measured using questionnaires developed for gaming and interactive media (e.g., the Immersive Experience Questionnaire, IEQ; Jennett et al., 2008), with medium-specific questionnaires also available for immersive virtual environments (Tcha-Tokey et al., 2016) and film/TV watching (e.g., the Film IEQ; Rigby et al., 2019). Researchers have also sometimes complemented self-report questionnaires with behavioral measures (e.g., voluntary engagement and willingness-to-pay; Hammond et al., 2023; Meyer, 2020) and, occasionally, physiological measurements (e.g., heart rate and skin conductance; Meehan et al., 2002; Rooney et al., 2012). Finally, “touch hunger” is understood as the innate human need for physical contact for psychological well-being (Field, 2014).

The focus on touch as a medium for collapsing the gap between fiction and reality is not new. As early as 1921, inspired by the societal changes following the First World War, Marinetti created his *Manifesto of Tactilism*. In this manifesto, the Futurist envisioned that physical touch could convey “continuous transmissions of thought” and deepen human connections and communication through tactile experiences (see Marinetti, 1921).^5^ Although Marinetti's tactile tables, art pieces that were designed as journeys for the hands using varied materials to evoke different sensations, never reached a wider audience, later scholars see Tattilismo as an early blueprint for today's immersive haptic media (Strauven, 2018).

Direct physical (i.e., interpersonal) touch can help to connect the performer with their audience by breaking down the separation between them, thus making the story feel more believable. If the storyteller's goal is to immerse the audience, then physical touch can be used to add an extra layer of realism, making it feel as though the story is actually happening and that the audience is physically part of the setting. However, the role of touch varies depending on whether it is initiated by the performer or the audience (although both can, of course, occur simultaneously). Here, physical touch refers specifically to direct interpersonal touch, where the performer and the audience engage in real physical contact. Note that the interpersonal physical touch discussed here is often referred to as “social touch” in the literature. Here, we use the term “physical touch” primarily to emphasize real-world contact, as opposed to digital touch. When the performers initiate touch, it often functions as an invitation into the narrative world, creating a sense of guided immersion and welcoming the audience into the experience. In contrast, audience-initiated touch can foster a sense of agency, encouraging self-exploration within the setting and giving the narrative a more interactive, game-like quality. In both cases, touch can play an important role in breaking the “fourth wall” between the audience and the narrative, transforming passive observation into active participation (Fisher, 2012). However, touch, no matter whether initiated by the performer, the audience, or both, may be more likely to break the “fourth wall” and to be experienced as enhancing immersion when it is narratively interpretable and coherently mapped to the narrative events. In a previous study, Gao et al. (2026) demonstrated that with semantically congruent, synchronized pressure feedback mapped to on-screen events, the participants reported that tactile feedback was easier to understand and felt more meaningful when integrated with the audiovisual context. Similarly, in the Disney case mentioned above, tactile feedback on its own often carries limited narrative meaning (though it may have affective value—e.g., as in the case of aromatherapy massage; Spence, 2003). However, it is more likely to be interpreted as narratively meaningful, even if it happens to be very simple.^6^

## The Dimensions of Physical Touch in Immersive Entertainment

Before discussing the specific dimensions of physical touch in detail, Table 1 summarizes the key examples of touch in immersive entertainment that are discussed in this review.

**Table 1.** A comparative summary of key examples of touch in immersive entertainment, analyzed by context, voluntariness, audience participation level, and type of touch (direct/mediated).

Example	Context	Voluntary / Forced	Audience Participation	Direct / Mediated
Imponderabillia	Performance art	Forced	Passive	Direct
Disneyland Meet-and-Greet	Theme park	Voluntary	Active	Direct
Touch Sanitation	Performance art	Voluntary	Active	Direct
Touching 1000 People	Performance art	Voluntary	Active	
Kentucky Fried Movie—Feel-A-Round*	Film	Forced	Passive	Mediated
The Rocky Horror Picture Show	Performance	Voluntary	Active	Partially mediated
Sleep No More	Immersive theater	Voluntary	Active	Direct
Tag & Sensation	Game	Voluntary	Active	Direct

*This touch encounter is a concept imagined for the film and did not occur as a real-world entertainment context.

Across the examples summarized in Table 1, several cross-example principles could help to clarify when and why physical touch may shape immersion and affect in immersive entertainment. First, physical touch may increase presence and embodied realism by reducing the perceived distance between the audience and the story world, making participation feel more immediate and real, though this depends on context and comfort. Second, touch is more likely to enhance immersion when it is narratively interpretable and coherently mapped to the events that happen to take place in the experience. Third, agency and consent are fundamental prerequisites for participation. Fourth, scarcity and selection can amplify the perceived value of touch-based encounters. Fifth, the perceived human quality of touch depends not only on physical parameters but also on source attribution and social meaning. Taken together, touch may be sufficient to enhance immersion under these conditions, but it is not always necessary, and it may be inappropriate when boundaries, interpretation, or comfort are misaligned.

### Breaking the Fourth Wall: Physical Touch as an Invitation to the Story

One of the primary functions of physical touch in immersive theater is its ability to break the “fourth wall,” thereby serving as a direct invitation for the audience to enter the narrative world. The “fourth wall” refers to the implied boundary between performers/characters and the audience (i.e., “the diegetic storyworld and real life”; Schlütz et al., 2020). “Breaking” it involves performers acknowledging the audience's presence and inviting participation in the storyworld (e.g., direct address, guided interaction, or audience decision-making). Importantly, such transitions do not require physical touch, they can also be achieved through nontactile interactive cues (such as, e.g., when olfactory cues present on stage can be smelled by the audience in the context of live performance; see Spence, 2021b). Nevertheless, when touch is diegetically integrated and consensual, it can provide a particularly powerful means of breaking this boundary and, by so doing, increase the sense of immediacy and embodied realism.

Perhaps one of the best examples of physical touch as an invitation into a story is found in the work of the immersive theater company Punchdrunk, whose signature one-on-one interactions are demonstrated in their famous piece, Sleep No More. Unlike traditional theater, Sleep No More blurs the boundaries between the audience and performers, creating a more engaging and interactive experience (Cartelli, 2020). During the show, the action unfolds across multiple rooms and floors simultaneously, allowing audiences to freely explore the spaces. One important mechanism among all the interactions that help to break the so-called “fourth wall” (Gordon, 2020) is the one-on-one interaction, a signature feature of the format (Hopkins, 2024). Here, performers might invite an audience member to dance, while others may be offered tea, or kissed on the cheek, and so forth.^7^

In such immersive theater settings, the one-on-one encounters are usually diegetic, meaning that the touch is an integral part of the narrative and directly contributes to the storytelling (Spence & Gao, 2024b). Unsurprisingly, audiences are eager for these one-on-one opportunities, which transform them from passive observers into active participants. According to a post from Cartelli (2020): “this time I got FIVE……I was euphoric!!!” While this anecdotal evidence suggests high audience value for these physical touch-based interactions, it is important to note that other factors, such as the performance's narrative design and the exclusivity of being “chosen,” may also contribute to the excitement. That said, the extent to which touch encounters causally enhance immersion or affect remains unclear and could be further investigated in future studies.

Physical touch can enhance the sense of realism by providing direct sensory feedback, making the narrative worlds feel more authentic. Through such physical engagement, audience members switch from a passive role to more of an active one, becoming part of the narrative, moving freely within the performance space, and interacting with the actors. Through direct physical touch, the audience or participants can immerse themselves more deeply in the environment, thus somehow reducing the perceived distance between the virtual and real worlds. Without touch, immersion may be weaker for some participants, and the environment may feel less vivid or tangible. The absence of tactile feedback can make the narrative world feel somewhat “hollow” or “unreal.” The integration of physical touch may therefore help to enhance presence and engagement, making the experience feel more vivid, immersive, and participatory. By engaging directly with performers or elements of the environment, participants may receive personalized and potentially memorable experiences that create intimacy. This direct interaction can help them to feel more involved in the narrative and may foster greater emotional and cognitive investment.

Overall, one-on-one interactions in the context of immersive theater highlight the potential power of physical touch in enhancing engagement, fostering intimacy, and transforming audience members into active participants. Note here, however, that the power of these one-on-one touch encounters is likely heightened by their scarcity, as only a small fraction of a large audience is typically “chosen” for such an intimate experience.

### Emotional Resonance: Physical Touch to Build Emotional Connection and Trust

Beyond breaking narrative barriers, physical touch serves an important role in establishing deep emotional resonance, fostering a powerful sense of connection and trust between the audience and the narrative. The use of direct touch is not limited to immersive theater. A similar, though distinct, form of interaction occurs in theme parks, where physical contact helps build emotional connections between fans and characters. This role is illustrated by the meet-and-greet experience previously mentioned at theme parks like Shanghai Disneyland with LinaBell. There, the reintroduction of hugs and handshakes was associated with a huge increase in queue times (Pan & Wang, 2023; Zou, 2023) and suggests how deeply the audience values this physical interaction.

The emotional resonance of this interaction and the connection it builds can be understood through two psychological concepts. First, it speaks to the idea of “touch hunger,” the fundamental human need for physical contact, the absence of which became particularly apparent after periods of social distancing (Field, 2014). The touch from LinaBell becomes a moment of satisfying this need, a practice sometimes referred to as the “Disney Hug Rule,” where performers don’t let go until the child does, inspired by Walt Disney's belief that “you never know how much that child may need that hug” (Minn, 2025). Second, the long queues demonstrate “positive consumer contagion,” whereby consumers place a higher value on experiences “touched” by a desirable entity (Argo et al., 2008). The physical contact allows for a perceived transfer of the character's magic onto the fan, making the brief interaction incredibly valuable. Here, it is important to acknowledge that the correlation between queue length and the reintroduction of physical touch obviously does not establish causality. Other factors, such as changes in postpandemic consumer behavior, the Disney character's specific characteristics, or social media promotion, may also have contributed to the increase in demand. Controlled comparison conditions would be necessary to isolate the specific contribution of physical touch.

Therefore, this is not just any touch, it is a tactile engagement that triggers powerful emotional resonance. Much like a child sitting on Santa's lap at Christmas, the physical interaction makes the narrative world tangible and believable (Godwin, 2017; Jones, 2020). In experiences such as touching Disney characters or animals, physical contact provides participants with a sense of involvement and purpose. When people touch real objects, their emotional responses (such as excitement, comfort, or surprise) are often stronger and more authentic than in comparable no-touch (visual-only) interactions and when touch is absent (Gallace & Spence, 2010, 2014; Hoffman et al., 1998; Zha et al., 2025). However, the quality of this emotional response is not random, research shows that it can be guided by the nature of the touch itself. Esling (2021) found that direct, skin-to-skin contact significantly heightened spectators’ sense of intimacy and improved narrative recall when compared with gloved or no-touch conditions. This conclusion was supported by a comparative one-to-one performance design, using immediate narrative recall alongside postexperience reflections. The results also suggest a boundary condition that the impact of touch depends on how it is framed in the narrative (if it is contextually meaningful) and sustained within the interaction. In addition, the intention behind the touch also plays an important role. For instance, specific touch gestures with clear emotional intentions (such as a “squeeze” for pleasantness or “finger touch” for comfort) directly influence how the touch is interpreted emotionally by the receiver/audience (Rantala et al., 2013; see Gallace & Spence, 2010, for a review of the experimental literature on interpersonal touch). Consequently, the entertainment experience becomes more layered, empathetic, engaging, and immersive when the intention of each touch is carefully considered and matched to the desired emotional outcome or narrative line. Ultimately, this reinforces the notion that in a narrative environment, “touch makes real” (Gallace & Spence, 2014), thus helping to create a memorable and lasting emotional connection. Note that the memorability of such encounters may also be amplified by their relative rarity in entertainment contexts.

### Active and Passive: Touch Initiation Builds Engagement and Agency

The role and impact of physical touch in immersive entertainment are also dependent importantly on who initiates the contact and whether or not it is voluntary. The spectrum of interaction ranges from forced, passive encounters to active, collaborative engagements, each shaping the audience's sense of agency differently, thus impacting the immersive experience. At one end of the spectrum, one might consider the performance art Imponderabilia (1977) by Marina Abramović and Ulay. In this performance, the two artists—who were married at the time—stood naked, facing each other in a narrow doorway, forcing audience members to physically squeeze between them if they wished to pass through.^8^ Unlike the optional and voluntary touch in Disneyland or Sleep No More, this interaction was mandatory and unavoidable. This forced contact evoked discomfort and awkwardness rather than enhancing engagement with a narrative. In addition, in many forms of digitally mediated touch, tactile stimuli are typically triggered by the system (e.g., preprogrammed or event-driven), whereas for interpersonal touch, it is often more important that the viewer chooses to engage (i.e., clear opt-in/opt-out), given the added social meaning and boundary implications of being touched by another person. The contrast raises two related questions: When physical touch is forced rather than voluntary, how does it affect immersion and engagement? Does touch enhance the experience because of the tactile sensation itself, or because the audience has the power to choose whether or not to engage?

In contrast to Imponderabilia, the work of Mierle Laderman Ukeles, “Touch Sanitation” (1979–1980) showcases a different form of performer-initiated touch, which instead creates recognition and agency. In this piece, Ukeles shook hands with over 8,500 sanitation workers across New York City, actively initiating touch to express gratitude and bridge social divides. While the workers were passive recipients of the handshake, the act was a voluntary social contract, bestowing recognition and agency through respectful contact.^9^

At the opposite end of the spectrum, we find active, participant-driven touch in modern gaming contexts. The Sensation project, a human-to-human touch project, is a control apparatus designed to detect different patterns of touch between two players, enabling a rich, collaborative experience through physical contact. In the game Shape Destroy, players use specific patterns of touching, such as a palm touch or a fist bump, to collaborate and complete tasks. Players are only able to progress in the game by touching each other. This method has been found to increase levels of engagement compared to using traditional game controllers such as gamepads, particularly in terms of shared emotional engagement (Canat et al., 2016a, 2016b). By incorporating various forms of touch such as hugging, fist bumps, and handshakes as the core game mechanic, experiences such as Musical Embrace and Shape Destroy make the digital experience physical (Canat et al., 2016a, b), grounding the virtual collaboration in a tangible and shared reality, intensifying the experience and making those moments even more memorable from a social perspective.

In summary, the question of who initiates touch is a fundamental differentiator in the design of immersive experiences that involve physical touch. While the collaborative touch in the Sensation project fosters shared engagement by maximizing player agency, the forced touch in Imponderabilia can be seen as transforming the experience into a social–psychological challenge by constraining agency once participants choose to proceed. Meanwhile, examples like Touch Sanitation demonstrate that even performer-initiated touch can grant agency and create positive connection when grounded in respect and voluntary participation.

Across the various examples reviewed here, it is important to distinguish between touching (initiating contact) and being touched (receiving contact). This review focuses on the audience perspective (i.e., being touched). Touch rarely acts on its own, narrative structure, novelty, being selected and memory-making can all help to shape the overall experience. We suggest that touch often functions as an embodied cue that shapes or amplifies the impact of these surrounding factors.

### The Boundaries and Future Between Physical Touch and Digital Touch

The distinction between human and nonhuman touch also affects the design of immersive experiences. In Marina Abramović and Ulay's Imponderabilia, the unpredictability and emotional ambiguity (and possibly uncomfortable nature) of the direct contact with the human performers contributed to the psychological tension of the piece. Had the performers been robots, or had the audience only experienced vibration instead of actual human presence, the intensity of the encounter would likely have been much diminished. The difference in hypothetical outcomes shows the limitation of artificial touch: its struggle to replicate the profound psychological tension generated by a real, ambiguous human presence (i.e., the copresent involvement of a real person whose intentions and the social meaning of the contact are not fully predictable due to human agency).^10^

In the context of VR, developers have explored “passive haptics” to bridge this gap between the virtual and the real. This involves placing real, physical objects in the user's space that correspond to objects in the virtual world. Early controlled experiments demonstrated the effectiveness of this approach. For instance, Hoffman et al. (1998) found that a “see and touch” group, who physically handled a real object corresponding to a virtual one, reported significantly higher levels of realism compared to a “no touch” group. However, it is important to note that this study was conducted with a small sample size (*N* = 19 in Experiment 1). As such, one might hope for larger-scale replication to confirm the generalizability of these findings. Similarly, Gallace et al. (2012) reviewed the literature showing that passive haptic setups could enhance presence and spatial awareness by grounding digital experiences in the physical world.

Despite its proven benefits, the passive haptics approach faces some limitations. Early studies such as Hoffman et al. (1998), as just mentioned, were based on small sample sizes (19 students in Experiment 1 and 21 students in a pilot study), which limits the generalizability of their findings. Moreover, these approaches are often constrained by scalability, technical complexity, and the immense challenge of replicating the nuanced qualities of interpersonal touch, such as warmth or subtle textures (Price et al., 2021).

An even more playful imaginary exploration of physical human touch appeared in the 1977 film The Kentucky Fried Movie, which imagined a “feel-a-round” cinema where human ushers would physically replicate on-screen events for the audience, hitting the audience, spraying them with perfume, giving them a massage, etc. This illustrates using human touch to enhance an immersive experience (a physical touch approach to today's 4DX cinema).

Compared to physical touch, digital touch offers flexibility and scalability, allowing it to easily expand or adapt across different applications and environments. This enables it to simulate non-realistic experiences and makes it applicable to a variety of scenarios. However, one of the most obvious limitations of most forms of digital touch is the lack of temperature and texture feedback, which are crucial in the case of affective touch. C-tactile afferents, sensitive to gentle, slow stroking at neutral skin temperature (∼32°C), are key to eliciting emotional responses, social bonding, and touch-based empathy (Ackerley et al., 2014; Morrison et al., 2011; Spence, 2022). While digital environments can enhance perceived sensuality through cognitive factors such as product labeling (McCabe et al., 2008) or background music (Fritz et al., 2017), replicating the richness of human touch remains challenging. This illustrates how affective touch depends not only on physical properties but also on cognitive framing, making it especially difficult to reproduce digitally.

In contrast, human touch offers warmth, emotional richness, and social meaning (Willemse, 2015) and can therefore shape affective responses and memory for experiences, depending on context and interpretation (e.g., Gallace & Spence, 2010, 2014; Morrison et al., 2011). For instance, physical touch has been shown to increase the generosity of shoppers (the “Midas touch effect”; Zha et al., 2025) or trigger excitement during theme park rides (see Spence, 2021a, for a review; cf. Spence, 2024). However, when delivered mechanically, this type of touch may not necessarily evoke the same emotional depth as direct human physical contact, such as a handshake or hug from a performer. That being said, in contexts such as Ghost Train rides, where the source of the touch is uncertain and ambiguous, the audience may misattribute any physical tactile sensation to being touched by a ghost, thus leading to different emotional responses as compared to when they are aware that any touch they feel comes from a machine or inanimate object (such as a “dangler,” the name given to the bits of cloth that hang down and touch the passengers’ faces as their trolley is moved through the dark environment). While both forms of touch can enhance presence, direct human touch, or the belief that it is direct human touch, often leads to more profound emotional responses, such as feelings of empathy and connection, which mechanical touch lacks. As Gallace and Spence (2014) note, proximity to another person, which allows for physical touch, also typically introduces additional sensory cues, such as smell, further intensifying emotional responses and enriching the sensory experience (see Table 1, presented earlier, for a summary).^11^

## Cultural Variations in the Acceptance of Physical Touch

Physical touch is not universally perceived in the same way across cultures, and it varies significantly among them, a phenomenon studied within the field of proxemics (Hall, 1966). One key factor is the perception of personal space. This aligns with Hall's classification of “contact cultures.” For example, in many Western cultures, maintaining a certain physical distance is a social norm, while in Mediterranean or Latin American cultures, closer proximity is often accepted in social interactions, and “non-contact cultures,” like many in Northern Europe and East Asia, are those where greater physical distance is the norm. This phenomenon also aligns with observations in European cafes and public spaces, where Southern European dyads stood closer and touched more than Northern European dyads (Remland et al., 1995), and with a 42-country survey (*N* = 8,943) documenting large cross-national differences in preferred distances toward strangers, acquaintances, and close others (Sorokowska et al., 2017). These behavioral patterns may be rooted in deeper cultural values, particularly the individualism–collectivism dimension (Hofstede, 2001), in which individualistic societies often place a higher premium on personal autonomy and space. Moreover, a person's psychological state is another key factor. For instance, when people experience stress or feel threatened, they tend to increase the distance between themselves and others (Dosey & Meisels, 1969), thus reducing the possibility of physical touch. Additionally, cultural norms dictate which body parts are considered appropriate to touch. In Thailand, for instance, the head is considered the most sacred part of the body, consequently, touching it is often regarded as disrespectful. Understanding these cultural nuances is crucial when designing physical touch experiences in global entertainment settings, as failing to do so might lead participants/audiences to feel that their personal space has been invaded, significantly affecting their comfort and emotional state (Felipe & Sommer, 1966; Horowitz et al., 1964).

## Limitations of Incorporating Human Touch in Immersive Entertainment

Despite its potential, incorporating physical touch into immersive entertainment presents several limitations. First, concerns around privacy, personal space, and ethical boundaries are important, particularly when touch is unexpected or occurs without explicit consent (Ley & Rambukkana, 2021). Similar issues extend to digitally mediated touch in intimate contexts (including adult entertainment), raising additional questions about consent, privacy, and platform governance. Audiences vary widely in terms of their comfort levels, cultural norms, and personal boundaries, requiring careful design and instructions to avoid discomfort or exclusion. Consent communication should be integrated into the preshow without necessarily undermining immersion, it may make the audience (and also the performers) feel safer and thus increase the pleasantness of the experience. Consent in immersive touch should be continuous, and designs must support opt-out and immediate disengagement without penalty (Ableson, 2018).

Second, cost, labour, and technical complexity can make the implementation challenging. Systems that enable synchronized or responsive touch, whether via human performers, wearable devices, or environmental actuators, often require financial and logistical investment. For example, only a small fraction of a theme park's visitors might receive personal character interaction on a busy day. To increase capacity, more staff must be hired, and greater investment in training and rehearsal is needed. There is also concern that once the novelty wears off, maintaining the same level of audience interest would require more effort over time. These challenges are also amplified by limited venue space, which may constrain movement or reduce opportunities for physical interaction.

Third, many touch-based experiences lack scalability, making them difficult to adapt to large audiences and, in practice, becoming financially challenging to operate (Alston, 2019). What works in an intimate installation or small-scale performance will likely simply not translate effectively to a theme park or commercial VR experience, such formats often sell a handful of tickets per night, limiting their impact and raising questions about sustainability (Alston, 2019). In addition, there remain substantial challenges in replicating the emotional depth of human touch through mechanical or digital means. As discussed earlier, physical properties such as temperature and pressure are difficult to simulate convincingly.

Fourth, physical touch in the context of public entertainment carries not just social risks but also health concerns. Close contacts such as hugs, high-fives, or handshakes can increase concerns about hygiene and infection risk, which may reduce participation. It is worth noting that post-COVID, people may be less willing to engage in touch-based interactions due to heightened hygiene and infection concerns (Ujitoko et al., 2022). On the other hand, clear and well-communicated hygiene practices can show care for guests’ well-being, build trust and comfort, and enhance their overall sense of security (e.g., Disney's COVID-era “yellow line” policy limiting physical contact, or immersive productions that suspended certain one-on-one touch interactions).

Finally, while most discussions of physical touch in immersive entertainment focus on performers touching the audience, there are also cases where the audience interacts physically with each other. For example, in religious settings, such as church services where congregants shake hands—a practice that was temporarily suspended during the pandemic restrictions but has since been reintroduced as restrictions eased (Reuters, 2022)—physical touch serves a social bonding function rather than a narrative-driven interaction. These interactions raise additional considerations regarding personal space, comfort levels, and cultural differences, which may affect audience engagement and the overall experience.

## Future Directions

### Mimicking Physical Touch in Digital Environments

Human touch consists of different elements, including temperature, movement, and the dynamic quality of stroking. Current haptic technologies, such as vibrotactile feedback and force feedback, can partially replicate these sensations but often lack the warmth and the nuanced movement found in real human touch (e.g., Po2 uses two vibrating actuators on the hands to render illusory tactile motion for gesture-based gameplay^12^; Israr et al., 2015). Further research could explore the differences between human touch and digitally simulated touch with movement and warmth.

In addition, although this review focuses on the audience experience of being physically touched in entertainment, the core principles (semantic intent and spatiotemporal specificity) from interpersonal touch may also help to inform the design of mediated or robotic touch (e.g., wearables, soft haptics, and affective human–robot interaction [HRI]). Soft and compliant interfaces may be especially promising for rendering more organic interpersonal contact (e.g., squeezes, hugs, and strokes) that are difficult to achieve with vibrotactile cues alone (Rognon et al., 2019; Yohanan & MacLean, 2012). For example, Disney's Avatar Flight of Passage integrates vibration, softness, and inflatable bladders in the ride seat to convey animal-like breathing and motion during flight (Walt Disney World Resort, n.d.). Touch can also convey affiliative emotions (App et al., 2011) and intent (Yohanan & MacLean, 2012). Thus, future haptic design may benefit from treating touch not only as a sensory signal but also as a design problem that requires mapping tactile feedback to narrative intent in time and space.

### Integrating Physical Touch with New Technologies (e.g., Mixed Reality [MR], Artificial Intelligence [AI])

Physical tactile feedback, when combined with MR and AI, can significantly enhance user immersion and perceived realism of virtual environments. By adding tactile feedback to virtual objects, users can more directly experience the physical properties of these objects, such as weight and texture, which increase the realism and naturalness of the interaction. This tactile engagement may help to make the virtual world feel more “real” and allows it to seamlessly blend with the real environment (Bhatia et al., 2024). Additionally, tactile feedback can enhance a user's sense of embodiment in an avatar, thus contributing to a more immersive experience. This can even reduce the perception of pain by enriching the virtual environment and increasing engagement within the virtual world (Hoffman et al., 2023). Furthermore, AI can personalize this process by continuously monitoring the user's behavior and adjusting the type and intensity of the tactile feedback accordingly. That said, there is also a danger that near-human but imperfect digital haptic encounters can evoke an “uncanny valley of haptics,” where cues are almost realistic yet perceptually mismatched, leading to eeriness and reduced presence (Berger et al., 2018).

### Exploring the Concept of Liveness in Transferred Physical Touch

Considering that Autonomous Sensory Meridian Response (ASMR) content can induce relaxation and a tingling sensation in viewers through audiovisual triggers, this ASMR response highlights the potential for touch experiences that do not rely solely on direct haptic feedback. Research suggests that ASMR triggers, such as whispering and precise soundscapes, can evoke tactile-like responses without physical contact (Barratt et al., 2017). However, a key distinction remains: While ASMR and other mediated sensory experiences can be replayed and shared widely, physical touch is inherently unique, ephemeral, and often limited to one or very few individuals at a time. These various issues raise questions about the authenticity and exclusivity of digital touch simulations and whether alternative sensory modalities can fully replicate the emotional and immersive impact of direct physical interaction. Future studies could, for instance, compare participants’ physiological and emotional responses while orthogonally varying haptic presence (live tactile encounter vs. no-touch ASMR) and liveness (live vs. prerecorded).

### Personalizing Physical Touch Experiences Based on User Preferences

In conclusion, it is important to remember that not all users respond to physical touch in the same way. Future directions should also take individual preferences including personal boundaries, cultural background, and past experiences, into consideration. Recent developments in adaptive systems and AI could potentially be used to tailor tactile feedback to individual users. By recognizing users’ individual preferences, physical touch can make the experience more immersive and engaging, provided it is inclusive, respectful, and emotionally effective.

Considering that tactile experience is not only a physiologically mediated perceptual experience but also a well-recognized channel for conveying emotion (Gallace & Spence, 2010, 2014), physical tactile feedback could be used in future immersive entertainment design to enhance and refine emotional experiences (e.g., a warm hug vs. scary tactile feedback, such as the sensation of something crawling on the skin; Spence, 2024).

## Notes

1. Some visitors even bring gifts for the Disney characters that the latter pretend to accept.

2. While increased park attendance was likely a factor, it may not fully account for the near tripling of the wait time.

3. Future work could, for example, evaluate this by contrasting skin-to-skin versus tool-mediated copresent touch on ratings of naturalness/realism, comfort/intimacy, and agency.

4. As noted by Gallace and Spence (2014), whenever one person is close enough to physically touch another, they are also close enough to smell the other, so olfactory and pheromonal cues may sometimes play a role in the multisensory experience of direct interpersonal touch as well (Croy et al., 2014; Croy et al., 2016).

5. In the 1930s, Salvador Dalí envisioned a “cinéma tactile” where audiences would be able to physically interact with objects corresponding to whatever was shown on screen. For instance, he proposed that viewers could touch rubber breasts while watching a scene of a woman being caressed or feel hot water sprayed on their hands as a bidet flushed in the film. However, such ideas were obviously not commercially feasible (see Spence & Gao, 2024a).

6. People sometimes remark on how simple the story behind LinaBell is compared to that of other Disney characters.

7. Though the emotional cost of providing all this tactile stimulation to the performers should not be neglected either (McDermott, 2013; Spence, 2017). Although Sleep No More provided a unique and immersive experience, it was criticized for lacking policies to prevent inappropriate touching by the audience in its early stages. The anonymity of the audience's masks may have further complicated the situation, as it potentially emboldened some individuals while also making it harder to monitor behavior. Under public pressure, the production later added a reminder for the audience to “keep a respectful distance” from the actors, but this measure was widely regarded as insufficient. In contrast, another immersive theater production, Then She Fell, implemented better policies: actors had the authority to end a scene if they felt uncomfortable, and security staff were trained to intervene immediately when necessary (Soloski, 2018).

8. Note that the intense psychological tension of the original 1977 performance is often considered inseparable from the fact that Abramović and Ulay were a real-life couple. When the piece was reproduced by other unrelated performers, for instance, during the 2010 MoMA retrospective “The Artist Is Present,” some critics observed that while the physical act remains the same, it lacks the tension that the original artists brought to the piece (Cumming, 2023). This observation highlights the fact that the perceived relationship between the individuals involved is an important variable in shaping the meaning and emotional impact of the tactile interaction.

9. A similar example, albeit one without interpersonal physical contact, was the UK's weekly “clap for carers” during the COVID-19 pandemic in 2020, which served a similar function of expressing public gratitude and recognition.

10. While physical touch has been extensively explored in the context of performance art, it is interesting to note that the International Society for Affective Touch (IASAT), despite its focus on the science of affective touch, does not, as far as we are aware, engage with performances that actually involve any form of interpersonal touch (International Society for Affective Touch, n.d.).

11. Studies have shown that scent can influence the pleasantness of touch and shape a person's perception of who is performing the touch.

12. Po2 demonstration: https://www.youtube.com/watch?v=Ybs4gsXfgwM
